# Treatment of Infected Tibial Metaphyseal Nonunions Using the Ilizarov Method: Protocol for a Prospective Nonrandomized Study

**DOI:** 10.2196/39319

**Published:** 2022-12-29

**Authors:** Konstantinos Sidiropoulos, Andreas Panagopoulos, Stelios F Assimakopoulos, Panagiotis Givissis, Antonios Kouzelis, Ioannis Vrachnis, John Lakoumentas, Alkis Saridis

**Affiliations:** 1 Emergency Department Papageorgiou General Hospital of Thessaloniki Thessaloniki Greece; 2 Medical School University of Patras Patras Greece; 3 Orthopaedic Department Patras University Hospital Patras Greece; 4 Department of Internal Medicine & Infectious Diseases Patras University Hospital Patras Greece; 5 Faculty of Medicine School of Health Sciences University of Patras Patras Greece; 6 Orthopaedic Department Aristotle University of Thessaloniki Thessaloniki Greece; 7 Department of Physics Patras University Hospital Patras Greece; 8 Orthopaedic Department General Hospital of Drama Drama Greece; 9 Orthopaedic Department General Hospital of Serres Serres Greece

**Keywords:** tibia, nonunion, infection, proximal metaphysis, distal metaphysis, Ilizarov, systematic review

## Abstract

**Background:**

The management of infected metaphyseal nonunion of the tibia is devastating, especially when associated with significant bone loss, poor soft tissues, draining sinuses, axial deformity, knee or ankle joint stiffness, limb discrepancy, and multiresisted pathogens. A systematic review, performed recently by the primary investigators but not yet published, yielded the lack of studies in the field and the huge heterogeneity of the presented results. We found several bias and controversies such as no clear definition of the exact part of the tibia where the nonunion was located, the pathogen causing the fracture-related infection, the number of previous interventions and time to presentation, and the exact type of treatment methods including the use of muscle flaps or bone grafting. Time to final union as a functional score is another important but missing data.

**Objective:**

The proposed study is designed to evaluate a sufficient number of patients with infected metaphyseal tibial nonunions using various general health, functional, and bone scores.

**Methods:**

This prospective clinical trial study, with a minimum follow-up period of 36 months, focuses on the effectiveness of the Ilizarov method after radical nonunion debridement and targeted antibiotic therapy in patients with infected metaphyseal tibial nonunions. The primary outcomes would be the definite healing of nonunion and infection-free results. Secondary outcomes would be limb alignment and discrepancy, alteration in the patient’s quality of life, and functional results. A power analysis calculated a minimum of 11 patients to obtain statistical power, but we aim to include at least 25 patients. Limb discrepancy, clinical validation of infection eradication and fracture healing, radiographic validation, and patient-reported outcome measures will be highlighted and correlated. Statistical analysis of the results will offer data missing from the literature so far. Measurements are scheduled at specific times for each patient: preoperatively, 3 and 6 months postoperatively, 1 month after Ilizarov frame removal, and once per semester afterward until the end of the follow-up period (minimum 36 months). Laboratory evaluation will be assessed once per month. Any complication will be reported and treated when it occurs.

**Results:**

The trial has already started. It was funded in June 2020. As of May 2022, 19 participants have been recruited and no major complications have been noticed yet. Data analysis will be performed after data collection ends, and results will be published afterward.

**Conclusions:**

An infected metaphyseal tibial nonunion is a rare condition with limited treatment options and many controversies. There is no consensus in the literature about the best treatment strategy, and this lack of evidence should be fulfilled.

**Trial Registration:**

International Standard Randomized Controlled Trial Number (ISRCTN) 30905788; https://www.isrctn.com/ISRCTN30905788

**International Registered Report Identifier (IRRID):**

DERR1-10.2196/39319

## Introduction

### Background

The infected nonunion of the tibia is always a challenging problem for the orthopedic surgeon and can pose a substantial burden on both patients and their families [1,2]. According to Schade et al [3], who performed a systematic review on 17,073 patients with open tibia fractures, the rates of infection, nonunion, and subsequent amputation were 22%, 11%, and 16%, respectively, with a total hospitalization cost between £356 (US $440) to £126,479 (US $156,313) and an average length of hospital stay of 56 days. Hendrickx et al [4], in their systematic review of 8110 patients treated with intramedullary nailing for a tibial shaft fracture, reported a nonunion rate of 11% and an incidence of early deep infection of 3%.

In a recent systematic review of 41,429 patients with tibial fractures, Tian et al [5] defined the main predisposing factors to nonunion: aged >60 years, male gender, BMI >40, smoking, diabetes, nonsteroidal anti-inflammatory drug or opioid use, fracture of the middle and distal tibia, high-energy fracture, open fracture, Gustilo-Anderson grade IIIB and IIIC, Müller AO type C, open reduction, fixation model, and infection. The authors found also that the prevalence of nonunion was 6.8% and that closed reduction and minimally invasive percutaneous plate osteosynthesis had the lowest risks of nonunion. Regarding the infection rates, a machine learning algorithm to identify patients with tibial shaft fractures at risk for infection after operative treatment was published recently, which identified seven stratified risks for infection: (1) Gustilo-Anderson or Tscherne classification, (2) bone loss, (3) mechanism of injury, (4) multitrauma, (5) AO Foundation/Orthopaedic Trauma Association (AO/OTA) fracture classification, (6) age, and (7) fracture location [6]. Metsemakers et al [7] tried to identify individual risk factors for both deep infection and nonunion or malunion after intramedullary nailing in the tibia and failed to identify any specific multifactorial model; polytrauma and primary external fixation were the only risk factors for nonunion and deep infection, respectively.

The incidence of infection and associated risk factors, especially for the fractured distal and proximal tibia metaphysis, are scarce in the literature. Parkkinen et al [8] reported a 5.2% incidence of deep infection (82% acute) on 655 proximal tibial fractures treated with open reduction and plate fixation; 50% required muscle flap coverage, and 5 patients (15%) eventually underwent above-the-knee amputation. The main risk factors included aged ≥50 years, obesity, alcohol abuse, AO/OTA–type-C fracture, and a previous fasciotomy. Bleeker et al [9] performed a systematic review on how we should personalize surgical treatment for the treatment of distal tibial fractures using either intramedullary nailing or plate fixation; 1332 patients were analyzed, including 10 randomized clinical trials (n=873) and 5 observational studies (n=459). Plating led to a lower risk for malunion but higher risk for infection (8%). No differences were detected regarding nonunion, subsequent reinterventions, and functional outcomes.

A narrative systematic review, registered on International Prospective Register of Systematic Reviews (PROSPERO; CRD42020205781) but not yet published, regarding septic metaphyseal tibial nonunion and their treatment strategy in adult populations, yielded the lack of studies in this field and the huge heterogeneity of the results. Moreover, through these studies, there was no clear definition of the exact part of the tibia where the nonunion was located, the pathogen causing the fracture-related infection (FRI) and nonunion, the number of previous surgeries and time to presentation, the exact type of treatment method, the use of muscle flaps or bone grafting, time to final union and eradication of infection, and finally, the report and management of complications. Above all, the absence of preoperative and postoperative functional and bone scores was the main cause that prevented us to extract safe conclusions about safety and efficacy. The limited number of patients (<100) with infected metaphyseal nonunions that were finally included in this review reflects the rarity and predicament of this condition.

### Aims of the Study

The proposed prospective study—Treatment of Septic Metaphyseal Nonunion of Tibia Using the Ilizarov Method (SePseT Ilizarov)—is designed to evaluate multiple clinical, radiological, and quality-of-life parameters in patients with infected tibial metaphyseal nonunions managed with the Ilizarov method and proper antibiotic treatment.

The *primary outcomes* of the study are bifold: (1) definite bone healing of the nonunion and (2) the absence of recurrent infection. The course of bony treatment will be further analyzed regarding complications and additional surgical interventions (before frame removal) until the end point, namely, healing or definite failure (amputation or death). The course of infection treatment will ascertain through the normalization of specific markers (C-reactive protein [CRP], erythrocyte sedimentation rate [ESR], and white blood cell count [WBC]), and the failure of antibiotic therapy will be defined as (1) recurrent infection with new positive cultures, (2) new sinus formation, (3) further surgical debridement, or (4) need for long-term antibiotic treatment for persistent symptoms.

*Secondary outcomes* will be the final limb length discrepancy (LLD), external fixation index (EFI), the Association for the Advancement of Methods of Ilizarov (ASAMI) bone and functional classification scores [10], and several patient-reported outcome measures including the American Orthopaedic Foot and Ankle Society (AOFAS) ankle-hindfoot score [11], the Knee Outcome Survey–Activity of Daily Living Scale (KOS-ADSL) score [12], the American Academy of Orthopaedic Surgeons (AAOS) Lower Limb Scale [13], the EQ-5D-3L [14], the quality-adjusted life year (QALY) Time Trade-Off [15], the Short-Form (SF) 12 and SF-6D [16,17], and finally, the 11-point Pain Numerical Rating Scale (PNRS) [18].

## Methods

### Trial Registration

The proposed study has been registered in International Standard Randomized Controlled Trial Number (ISRCTN; 30905788).

### Ethics Approval

This study was approved by the Ethics Committee of General Hospital of Serres (No 09/21-09-2020), General Hospital of Drama (No 336/2020, 04/09/2020), and University of Patras (No 5141/38886, 20-11-2020).

### Patient Consent

Informed consent will be taken from all the patients. We will obtain written consent to publish from the participants to report individual patient data.

### Design

We will enroll patients aged >18 years with infected metaphyseal nonunion of the proximal or distal tibia demonstrating the absence of healing for longer than 9 months and no observation of healing during the previous 3 months. Consent forms will be freely signed by the participants after a thorough explanation provided by lead investigators, and confidentiality is guaranteed according to General Data Protection Regulation rules. Patients will be excluded if (1) there is adjacent knee or ankle joint infection, (2) the bony defect once debrided exceeds 7 cm, (3) the foot is insensate, (4) there is a pathological fracture, and (5) there is evidence of hormonal disorders or diseases that affect bone healing.

### Evaluation

At presentation, the details and type of initial injury; previous surgical interventions and medical treatments; associated illnesses; other injuries; nicotine, alcohol, and drug abuse; and the affirmation of infection according to FRI criteria [19,20] will be documented. These FRI criteria can be either *confirmatory*—(1) fistula, sinus or wound breakdown; (2) purulent drainage from the wound or presence of pus intraoperatively; (3) phenotypic confirmation of the existence of the germ in at least two different deep tissue cultures; or (4) presence of microbes in deep tissue taken intraoperatively, as confirmed by histopathology—or *suggestive*, such as (1) clinical signs (pain, local redness, local swelling, increased local temperature, and fever >38.3 °C); (2) radiological signs (osteolysis, implant loosening, sequestration, bone healing arrest, and periosteal bone formation); (3) presence of pathogenic microorganism in a culture from the deep layer; (4) elevated inflammatory markers (ESR, WBC, and CRP); (5) persistent wound drainage after the first days, increasing or new onset; and (6) new onset of joint effusion in patients with fractures.

During physical examination, the patients will be screened for LLD, ankle and knee range of motion, pathological motion at the fracture site, neurovascular deficiency, and the condition of soft tissues. Long leg standing radiological views, computed tomography or magnetic resonance imaging if indicated, and 3-phase bone scintigraphy will be ordered to establish nonunion, bone defect, and osteomyelitis. Regarding bone deficit, the nonunion would be classified using the criteria of Paley [10] into type A when the defect is <1 cm (A1: atrophic flexible; A2-1: hypertrophic stiff, without deformity; and A2-2: hypertrophic stiff, with deformity) or type B when the defect is >1 cm (B1: bone deficit without shortening; B2: bone deficit and shortening without the dimension of the descendants; and B3: bone deficit and shortening).

### Intervention

After careful preoperative planning, the patients will be informed about the scheduled treatment plan and give their informed consent. Intraoperatively, all previous implants will be removed, and a radical debridement of avascular or infected bone and adjacent necrotic soft tissues will be performed such that bleeding bone ends will remain. The application of the frame will follow the well-established principles of Ilizarov with various ways depending on the degree of deformity: monofocal compression, monofocal distraction, bifocal acute compression, and gradual distraction or bone transport. Corticotomy will be performed proximally or distally, as indicated by the location of nonunion. A healthy soft tissue envelope will be achieved either with direct skin closure or using local muscle flaps; at least 5 samples of deep tissues will be obtained for culture and histopathology. The Cierny classification [21] will be used to classify the type of osteomyelitis: Type I (medullary), Type II (superficial), Type III (localized full-thickness cortical involvement), and Type IV (diffusely involves the entire circumference of a segment of the bone). All patients will discontinue any previous antibiotic therapy for at least 14 days before surgery to aid microbiologic diagnosis. Broad spectrum antibiotics (vancomycin-meropenem) will be administered intraoperatively followed by culture-specific antimicrobial therapy for at least 6-8 weeks. After the application of the Ilizarov frame, patients will be closely monitored every week and gradually every month in the outpatient clinic of the mentioned hospital. Distraction will be started on the seventh day at the rate of 1 mm/day, with 4 increments of 0.25 mm each day. Patients will be allowed to have full weight bearing with crutches for the first postoperative day, and early range of motion of the adjacent joints and muscle-strengthening exercises will be encouraged to prevent contractures.

### Power Analysis and Statistical Methods

**Figure 1:**
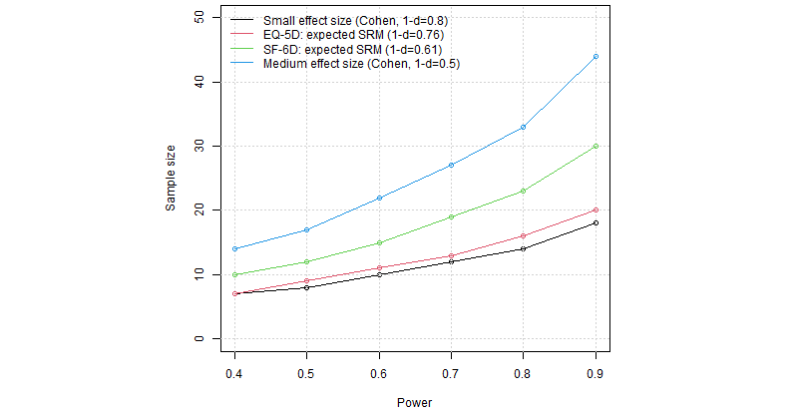
Power analysis—"distance to health" SRM: the statistical power of the study and number of the requiring patients for inclusion in the study. SF: Short-Form; SRM: standardized response mean.

To calculate the sample size of this study, we obtained information from the study of Jayadevappa et al [22] who assessed the usability of minimal important difference (MID) and minimal clinically important difference for measuring meaningful changes in disease-specific and generic health-related quality-of-life outcomes. Our primary outcomes, fractures healing and infection eradication, are not countable. As our patients will have different sites of nonunion (proximal or distal), the joint-specific outcome instruments (AOFAS and KOS-ADSL) will be not feasible. Instead, generic health-related quality-of-life outcomes will be more appropriate, namely, SF-6D and EQ-5D; the SF-6D is scored on a 0.29 to 1.00 scale and the EQ-5D on a –0.59 to 1.00 scale, with a score of 1.00 on both indicating “full health.” In the study of Jayadevappa et al [22], the mean MID for the SF-6D was 0.041 (range 0.011-0.097) and the mean MID for the EQ-5D was 0.074 (range –0.011 to 0.140). We assume that the continuous data are parametric—that is, all scores (questionnaire response) will follow a normal distribution; thus, Student 2-tailed *t* test can be applied. According to Walters et al [23], the scores are mainly in the “small to moderate” range using the criteria of Cohen [24] regarding the standardized response means (SRMs) of the questionnaire responses. Cohen’s criteria (in context of the one-sample *t* test) defines a small effect size at around d=0.2 and a medium effect size at around d=0.5, with d being the standardized difference of the means. We are mainly concerned with the comparison of the actual SRMs to the value 1, denoting “full health.” We call this concept “distance to health,” and in terms of statistical power analysis, we approach it with a one-sample *t* test, which compares an SRM to 1. Statistical significance was taken at <.05 by default, and the results are summarized in Figure 1. The implementation was held with the R software (package *pwr*; R Foundation for Statistical Computing) and the RStudio integrated development environment; both are open-source products. The required number of patients is from 20 to 30 for a power of 90% (Figure 1 and Table 1).

**Table 1:** Power analysis—table of data.

	Power (%), number of patients					
	40	50	60	70	80	90
Small effect size (Cohen, 1 – d = 0.8)	7	8	10	12	14	18
EQ-5D: expected SRM^a^ (1 – d = 0.76)	7	9	11	13	16	20
SF-6D^b^: expected SRM (1 – d = 0.61)	10	12	15	19	23	30
Medium effect size (Cohen, 1 – d = 0.5)	14	17	22	27	33	44

^a^SRM: standardized response mean.

^b^SF: Short-Form.

Values for subjective and objective outcomes will be presented as means with SDs or ranges. The outcome variables are union versus nonunion, the recurrence of infection or not, PNRS, ASAMI criteria, LLD, time to fracture healing, EFI, and the comparison between preoperative and final outcome scores (joint specific and generic). To deal with a set of scores (questionnaire responses) measured longitudinally, assuming normality, repeated measures ANOVA will be used to assess a comparison among the consecutive (paired) distributions. Bivariate comparisons will need to use Student paired 2-tailed *t* test. Prospective factors that have an effect on the aforementioned trajectories will be assessed with analysis of covariance; an alternative solution of the latter will be to use ANOVA on the differences of the scores (after – before).

### Outcome Assessment

The patient’s clinical, radiological, and functional outcomes at the end of the study period (minimum 3 years after the frame application) will be compared to the preoperative values. The main outcomes would be the definite healing of the nonunion and the eradication of infection. Inability to control the infection, joint arthrodesis, amputation, and death would be recorded as failures.

Various patient-reported outcome scores will be assessed preoperatively and at predetermined time intervals until the final outcome (Table 2). For the subjective clinical outcome, we intent to use 2 joint-specific scores, the AOFAS ankle-hindfoot score [11] and the KOS-ADSL score [12], and several general health questionnaires including the AAOS Lower Limb Scale [13], the EQ-5D-3L [14], the QALY Time Trade-Off [15], the SF-12 (physical and mental component scores) and SF-6D [16,17], and the PNRS [18]. For the functional outcome, we will use the LLD, EFI, and ASAMI bone and functional classification scores [10]. The latter is scored as excellent, good, fair, and poor. An excellent *bone result* equals to union, no infection, deformity of less than 7°, and tibia discrepancy <2.5 cm, whereas an excellent *functional result* means an active individual without limp, equinus rigidity, soft-tissue dystrophy, and pain. Finally, a detailed laboratory testing of kidney and liver function will be performed tactically in cases with prolonged antibiotic therapy. Protentional subgroups could be formed according to demographics, anatomic location, and functional scores. Trial results will be published after the end of the trial to enrich literature about this topic.

**Table 2:** Timetable and description of the preoperative and follow-up evaluation.

Outcome measure		Definition	Score	Preoperative evaluation	3-month follow-up	6-month follow-up	1 month after Ilizarov removal	Every 6 months up to the end of follow-up period (minimum 3 years)
**Patient-reported**								
	PNRS^a^	Pain Numerical Rating Scale	0-10	✓	✓	✓	✓	✓
	AOFAS^b^ ankle-hindfoot score	Foot and ankle–specific score	0-100	✓			✓	✓
	KOS-ADLS^c^	Knee-specific score	0-70 and 0-55	✓			✓	✓
	AAOS^d^ Lower Limb Scale	General lower limb condition	0-80	✓			✓	✓
	EQ-5D-3L	Health-related quality of life	5-15 (less the better)	✓	✓	✓	✓	✓
	QALY^e^ Time Trade-Off	Quality of life	N/A^f^	✓			✓	6 months after Ilizarov removal
	SF-12^g^ and SF-6D	General health questionnaire and QALY from the SF-12	Physical and mental component scores	✓	✓	✓	✓	6 months after Ilizarov removal
**Objective measures**								
	Limb discrepancy	Limb length normalization is one of the goals (fracture healing and infection eradication are the others)	N/A	✓	✓	✓	✓	✓
	Radiographic evaluation of bone healing	Fracture union and restoration of bone axis	N/A	✓	Every month	Every month	Every month	✓
	External fixation index	Time in external fixation / length of bone regenerated (months/cm)	N/A		After Ilizarov removal	After Ilizarov removal	After Ilizarov removal	
	Laboratory infection markers (CRP^h^, WBC^i^, ESR^j^, liver and kidney function)	Comorbidities and side effects due to the antibiotic therapy	N/A	✓	Every month	Every month	Every month	✓
	ASAMI^k^ scoring system (bone and functional)	Bone quality and functional results	Excellent, good, fair, or poor	✓			✓	✓
**Complications**		N/A	N/A	Intraoperative	Reported each and any time they appeared	Reported each and any time they appeared	Reported each and any time they appeared	Reported each and any time they appeared

^a^PNRS: Pain Numerical Rating Scale.

^b^AOFAS: American Orthopaedic Foot and Ankle Society.

^c^KOS-ADLS: Knee Outcome Survey- Activity of Daily Living Scale.

^d^AAOS: American Academy of Orthopaedic Surgeons.

^e^QALY: Quality Adjust Life Year.

^f^N/A: not applicable.

^g^SF: Short-Form.

^h^ASAMI: Association for the Study and Application of the Methods of Ilizarov.

^i^CRP: C-reactive protein.

^j^WBC: white blood cell count.

^k^ESR: erythrocyte sedimentation rate.

### Complications

Throughout the study period (3 years), all type of complications would be recorded and treated accordingly. These might include superficial pin tract infection, broken pins, transient knee or ankle flexion contracture, skin invagination or necrosis, equinus requiring Achilles tendon lengthening, ring fixator intolerance, nonunion of the docking site, refracture, late deformity of the regenerate callus, residual angular deformity, and residual LLD >2.5 cm. Any of these complications will be treated and reported accordingly. Due to the short period of the trial and the common risks of this intervention being established conditions, a data monitoring committee is not mandatory.

## Results

The trial has already started. It was funded June 2020. As of May 2022, 19 participants have been recruited. Data are collected on prescheduled dates according to the protocol’s timeline (Table 2), with the exception of complications, which are dealt with and recorded when encountered (no major complications have been noticed yet as of May 2022). Data analysis will be performed after data collection ends (ending time point is a minimum follow-up period of 36 months for all participants), and results will be published afterward.

## Discussion

### Overview

At least 25 patients will be recruited in this trial. These individuals will be followed up for a period of at least 36 months regarding not only fracture healing and infection eradication but also some other secondary outcomes; AOFAS ankle-hindfoot score, KOS-ADSL, AAOS Lower Limb scale, EQ-5D-3L, SF-12, and QALY Time Trade-Off will be assessed as patient-reported parameters. Objective measures, namely, limb discrepancy and its pre- and postoperative difference; laboratory infection makers (CRP, WBC, ESR, and liver and kidney function) and their alterations; and ASAMI (bone and functional), clinical (weight-bearing ability), and radiographic evaluation of fracture healing will be also assessed according to a preformed timetable (Table 2). As mentioned before, with any complication will be dealt with when they occur.

The statistical analysis of these parameters will provide valuable conclusions and proofs about the effectiveness and usefulness of the Ilizarov method during the treatment of septic tibial metaphyseal nonunions.

### Expected Findings

At the end of this trial, we hope to see fracture healing and infection eradication for all participants. Furthermore, a significant improvement of all patient-reported outcome measures is expected. ASAMI scores, both functional and bone, should be for the vast majority at least good, and LLD should ideally be absent or close to zero. In other words, our objective is patients returning to their work and social life with a healed, infection-free tibia.

The treatment of an infected tibial nonunion entails a substantial amount of time and patient discomfort. The first step in the work-up of these cases is a well-established diagnosis. An internationally accepted definition of FRI has been recently adapted [19,20], including 2 levels of certainty around diagnostic criteria: confirmatory (infection is definitely present) and suggestive (further investigation is required to exclude the possibility of an FRI) as previously described. Except for proper antibiotic treatment, the key aspects of surgical management are a thorough debridement, irrigation, fracture stability (usually with Ilizarov frames), dead space management, and adequate soft tissue coverage, but these approaches are usually compromised by the poor soft tissue status, active draining sinuses, osteomyelitis, osteopenia, LLD, and stiffness and contractures of the adjacent joints [25-29].

The problem is even more difficult when the infected nonunion is located to the proximal or distal metaphysis. The porosity of the cancellous bone differs from that of the cortical one because of the differences in cellularity, rich blood flow, and increased contact area. Therefore, the occurrence of metaphyseal nonunion is much more uncommon but more troublesome to treat, as it is often accompanied by poor bone stock (osteoporosis), small metaphyseal bone segment, deformity, bone deficit, soft tissue lesions, and posttraumatic arthritis [30-32].

The literature is scarce regarding infected metaphyseal tibial nonunions. For example, Eralp et al [33] and Brinker and O’Connor [34] used Ilizarov frames in infected distal metaphyseal nonunions of the tibia in compression or bone transport mode in combination with antibiotic therapy and reported good results. Siboni et al [35] and Yoon et al [36] reported very good results using the induced membrane (Masquelet) technique in combination with internal or external fixation and antibiotic therapy. A narrative systematic review, performed by the primary investigators but not yet published, yielded the lack of studies in the field and the huge heterogeneity of the presented results. We found several bias and controversies, such as no clear definition of the exact part of the tibia where the nonunion was located, the pathogen causing the FRI, the number of previous interventions and time to presentation, the exact type of treatment methods including the use of muscle flaps or bone grafting, the time to final union and eradication of infection, and finally, the report and management of complications. Above all, the absence of preoperative and postoperative functional and bone scores was the main cause preventing us to extract safe conclusions about the preferable treatment method.

### Limitations

The lack of control group, the location of the nonunion (proximal or distal), the size of bone defect, the condition of soft tissues, the heterogeneity of previous surgeries and implants, the different pathogens causing the infection, the length of the antibiotic therapy and frame application, as well as the different socioeconomic and psychological status of the included patients are some of the limitations of this study. However, its strongest advantage is the prospective design with the adequate number of participants and the multimodal evaluation with joint-specific and general health scores, including QALY (Time Trade-Off and SF-6D), as opposed to what already exists in literature.

### Conclusion

An infected metaphyseal tibial nonunion is a rare condition with limited treatment options and many controversies. There is no consensus in the literature about the best treatment strategy, and this lack of evidence should be fulfilled.

## Abbreviations

AAOS: American Academy of Orthopaedic Surgeons
AO/OTA: AO Foundation/Orthopaedic Trauma Association
AOFAS: American Orthopaedic Foot and Ankle Society
ASAMI: Association for the Study and Application of the Methods of Ilizarov
CRP: C-reactive protein
EFI: external fixation index
ESR: erythrocyte sedimentation rate
FRI: fracture-related infection
ISRCTN: International Standard Randomized Controlled Trial Number
KOS-ADLS: Knee Outcome Survey–Activity of Daily Living Scale
LLD: limb length discrepancy
MID: minimal important difference
PNRS: Pain Numerical Rating Scale
PROSPERO: International Prospective Register of Systematic Reviews
SePseT Ilizarov: Treatment of Septic Metaphyseal Nonunion of Tibia Using the Ilizarov Method
SF: Short-Form
SRM: standardized response mean
QALY: quality-adjusted life year
WBC: white blood cell count

## Data Availability

All data generated or analyzed during this study are included in the published article (and its additional files). Investigators will have limitless access to the final trial data set.

Trial results will be published after the end of the trial to enrich literature about this topic. All data will be available afterward.
